# Picomolar Dichotomous Activity of Gnidimacrin Against HIV-1

**DOI:** 10.1371/journal.pone.0026677

**Published:** 2011-10-24

**Authors:** Li Huang, Phong Ho, Jie Yu, Lei Zhu, Kuo-Hsiung Lee, Chin-Ho Chen

**Affiliations:** 1 Surgical Science, Department of Surgery, Duke University Medical Center, Durham, North Carolina, United States of America; 2 Natural Products Research Laboratories, Eshelman School of Pharmacy, University of North Carolina, Chapel Hill, North Carolina, United States of America; 3 Chinese Medicine Research and Development Center, China Medical University and Hospital, Taichung, Taiwan; New York Blood Center, United States of America

## Abstract

Highly active antiretroviral therapy (HAART) has offered a promising approach for controlling HIV-1 replication in infected individuals. However, with HARRT, HIV-1 is suppressed rather than eradicated due to persistence of HIV-1 in latent viral reservoirs. Thus, purging the virus from latent reservoirs is an important strategy toward eradicating HIV-1 infection. In this study, we discovered that the daphnane diterpene gnidimacrin, which was previously reported to have potent anti-cancer cell activity, activated HIV-1 replication and killed persistently-infected cells at picomolar concentrations. In addition to its potential to purge HIV-1 from latently infected cells, gnidimacrin potently inhibited a panel of HIV-1 R5 virus infection of peripheral blood mononuclear cells (PBMCs) at an average concentration lower than 10 pM. In contrast, gnidimacrin only partially inhibited HIV-1 ×4 virus infection of PBMCs. The strong anti-HIV-1 R5 virus activity of gnidimacrin was correlated with its effect on down-regulation of the HIV-1 coreceptor CCR5. The anti-R5 virus activity of gnidimacrin was completely abrogated by a selective protein kinase C beta inhibitor enzastaurin, which suggests that protein kinase C beta plays a key role in the potent anti-HIV-1 activity of gnidimacrin in PBMCs. In summary, these results suggest that gnidimacrin could activate latent HIV-1, specifically kill HIV-1 persistently infected cells, and inhibit R5 viruses at picomolar concentrations.

## Introduction

Human immunodeficiency virus type I (HIV-1) is the retrovirus that causes acquired immunodeficiency syndrome (AIDS). The AIDS pandemic is a serious public health problem for many countries in the world. Many drugs have been developed for AIDS therapy. The highly active antiretroviral therapy (HAART) that combines 3 to 4 anti-retrovirals has been successful in controlling HIV-1 replication in infected individuals. HAART has been shown to reduce plasma viral loads to undetectable levels in many HIV-1 infected patients [1], [2]. Although HAART can effectively control plasma viremia in many patients, the virus is suppressed rather than truly eradicated [3]–[6]. Persistent HIV-1 infection, especially in viral reservoirs, remains a challenge for effective AIDS therapy. In addition, other drawbacks, such as toxicity and side effects, often compromise the effectiveness of HAART. Thus, development of treatment regimens using novel drugs with potential to eradicate HIV-1 from its reservoirs is a major goal of current AIDS therapy.

**Figure 1:**
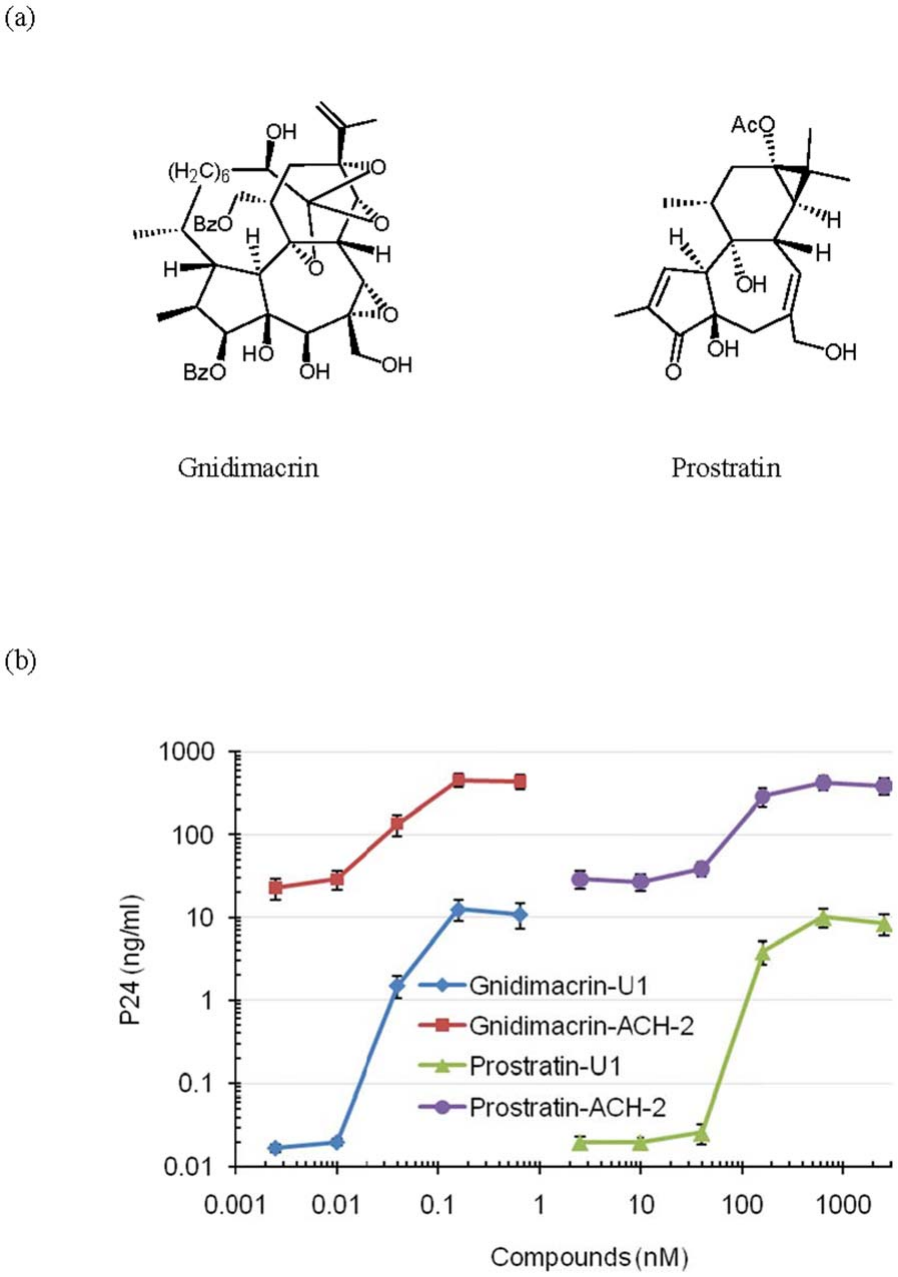
Gnidimacrin activated HIV-1 production in chronically infected cells at picomolar concentrations. (a) Chemical structures of gnidimacrin and prostratin. (b) Activation of HIV-1 production by gnidimacrin and prostratin. ACH-2 or U1 cells were treated with gnidimacrin or prostratin at various concentrations for two days. Each data point in the figure represents the mean +/− standard deviation of three independent experiments.

Daphnane diterpenoids are natural products with various biological activities [7]. Highly oxygenated daphnane diterpenoids were shown to inhibit HIV-1 infection at low micromolar concentrations [8], [9]. Gnidimacrin is a daphnane diterpene that can be isolated from different plants in *Thymeleaceae* [10]–[14]. It exhibited potent anti-cancer cell activity through activation of protein kinase C. Gnidimacrin shares some structural similarity to other anti-HIV-1 diterpenes, such as prostratin (12-deoxyphorbol-13-acetate), DPP (12-deoxyphorbol-13-phenylacetate), and ingenol derivatives (Figure 1a). Prostratin is a tigliane diterpene that was well documented for its anti-HIV-1 activity [15]–[17]. Prostratin is a non-tumour promoting phorbol ester that can inhibit HIV-1 infection, and activate HIV-1 replication in latent infection cell models. Thus, prostratin has been considered as a drug candidate to purge HIV-1 from latent reservoirs. Similar to prostratin, DPP is also a tigliane diterpene that was shown to potently inhibit HIV-1 at nanomolar concentrations [18]. In addition to the tigliane diterpenes, ingenane diterpene derivatives were reported to have anti-HIV-1 activity comparable to that of DPP [19], [20]. The anti-HIV-1 activity of these compounds was, at least in part, due to their ability to activate protein kinase C and down-regulate HIV-1 receptors, CD4 and chemokine receptors [16]–[20].

We have recently discovered that daphnane diterpenes, including gnidimacrin, exhibited potent anti-viral activity against the ×4 virus NL4-3 replication in MT4 cell lines [21]. However, their effect against HIV-1 primary isolates, mechanism of action, and ability to activate latent HIV-1 remain to be determined. Among the daphnane diterpenes, gnidimacrin was chosen for this study because it has been well characterized for its anti-cancer cell activities [10]–[14]. This study demonstrates that gnidimacrin activated HIV-1 production from chronically infected cell lines, ACH-2 and U1, at picomolar concentrations. In addition, gnidimacrin is a low picomolar HIV-1 R5 virus inhibitor that is thousands fold more potent than prostratin. Prostratin is currently the most well studied leading protein kinase C activator for adjuvant therapy toward HIV-1 eradication. Our results suggested that gnidimacrin targets protein kinase C beta to down regulate CCR5 and CD4 for the potent anti-HIV-1 R5 activity.

## Materials and Methods

### Materials

All the HIV-1 primary isolates used in this study were obtained from the NIH AIDS Research and Reagent Program. The anti-CD4 mAb OKT4 was provided by Dr. Celia LaBranche, Duke University, NC. The anti-CXCR4 mAb 12G5 and the anti-CCR5 mAb clone 45531 were obtained from the NIH AIDS Research and Reagent Program for FACS analyses. Gnidimacrin is provided by Dr. Wei Li, Toho University, Japan. Prostratin was purchased from LC Laboratories, Woburn, MA. The protein kinase C inhibitor, enzastaurin, was purchased from LC laboratories, Woburn, MA. ACH-2, U1, U937, and MT4 cell lines were obtained from the NIH AIDS Research and Reagent Program. Human PBMCs were prepared from whole blood obtained from the American Red Cross, Charlotte, NC.

### Antiviral assay

Anti-HIV activity of the tested compounds against NL4-3 infection of MT4 cells was determined using a p24 production assay. To determine anti-viral activity, the virus at a multiplicity of infection (MOI) of 0.001 TCID50/cell was used to infect MT4 cells in the presence of various concentrations of the compounds. On day four post-infection, culture supernatant was harvested and assayed for p24 using an ELISA kit from PerkinElmer. Drug concentration that inhibits HIV-1 p24 production by 50% is defined as the EC_50_ of the compounds. EC_50_ was determined with a non-linear regression analysis using the Biosoft software, Calcusyn.

### Determination of the anti-HIV-1 activity against primary isolates

A peripheral blood mononuclear cell (PBMC) assay was used to evaluate the antiviral activity of the tested compounds against a panel of HIV-1 primary isolates [22]. Human PBMCs were isolated from whole blood and CD8 cells were depleted by using Dynabeads purchased from Invitrogen. The CD8-depleted phytohemagglutinin (PHA) stimulated PBMCs were infected with virus at a multiplicity of infection (MOI) of 0.001 TCID_50_/cell in the presence of various concentrations of the compounds. The infected cells were cultured at 37°C for four days at which time 50% of the culture suspension was removed and fresh medium containing appropriate concentrations of the compounds was added. The culture supernatants were collected at day seven post-infection for p24 assays.

### Determination of the effect of enzastaurin on the anti-HIV-1 activity of gnidimacrin

To determine whether PKC beta plays a role in the anti-HIV-1 activity of gnidimacrin, CD8-depleted PHA activated PBMCs were pre-treated with various concentrations of enzastaurin for 2 hours. The enzastaurin treated PBMCs were then infected with HIV-1 BaL in the presence of various concentrations of enzastaurin and 0.1 nM of gnidimacrin or 0.5 uM of prostratin in 100 ul of RPMI medium supplement with 20% FCS, IL-2 (10 U/ml), penicillin (100 U/ml), and Streptomycin (100 U/ml). Fresh medium (100 ul) containing appropriate reagents was added to each well on day 4 post infection. The culture supernatants were assayed for p24 on day 7 post infection.

### Determination of the cytotoxicity of the compounds

Compounds were tested for cytotoxicity against different cell types. Cell lines at 1×10^5^ cells/ml, or PHA-activated PBMCs at 1×10^6^ cells/ml, were added to each well in a 96-well plate in the presence of various concentration of the tested compounds for an indicated period. Cell viability was determined by using a Promega cytotoxicity assay kit, CellTiter-Glo® Luminescent Cell Viability Assay, following the manufacturer's instruction. The drug concentration that results in a 50% decrease in viable cells is defined as the IC_50_ of a compound. The selectivity of a compound (selectivity index or therapeutic index) is defined as IC_50_/EC_50_.

### FACS analysis of HIV-1 receptors

MT4 cells or PBMCs were treated with gnidimacrin or prostratin for various times. The cells were then incubated for 30 min on ice with primary antibodies against CD4, CXCR4, or CCR5 in phosphate buffer saline (PBS) with 1% fetal bovine serum (FBS). The primary antibody was removed by washing the cells twice in PBS with 1% FBS. These cells were incubated for 30 min on ice with FITC-conjugated secondary antibody (BD Biosciences, WI). Cells were washed three times and fixed with 1% formaldehyde-PBS before analysed with a FACSCaliber cell sorter (Becton-Dickinson, CA). The level of each receptor on cell surface was expressed as % control which is defined as 100× (MFI_c_-MFI_b_/MFI_0_-MFI_b_), where MFI_0_ is the mean fluorescence intensity in the absence of a compound, MFI_c_ is the mean fluorescence intensity in the presence of the test compounds, and MFI_b_ is the background mean fluorescence intensity when the primary antibodies were not used in the assays.

## Results

### Gnidimacrin potently activated HIV-1 production from chronically infected cells

ACH-1 and U1 cells were HIV-1 chronically infected cell lines that have been used as *in vitro* cell models for HIV-1 latent infection [23], [24]. U1 cells were derived from the monocytic U937 cells chronically infected with HIV-1 and ACH-2 cells were HIV-1 chronically infected T cells derived from the lymphoblastoid cell line CEM. The cells were treated with gnidimacrin or prostratin at various concentrations for two days. In agreement with previous reports [16], [17], the non-tumour promoting phorbol ester prostratin activated HIV-1 production in both ACH-2 and U1 cells at sub-micromolar concentrations (Figure 1b). On the other hand, gnidimacrin activated HIV-1 production from both cell lines at picomolar concentrations (Figure 1b). Although both prostratin and gnidimacrin can activate HIV-1 production from these latent HIV-1 infection model cells, gnidimacrin is at least 2,000-fold more potent than prostratin.

### Gnidimacrin potently inhibited NL4-3 infection of MT4 cells at picomolar concentrations

**Figure 2:**
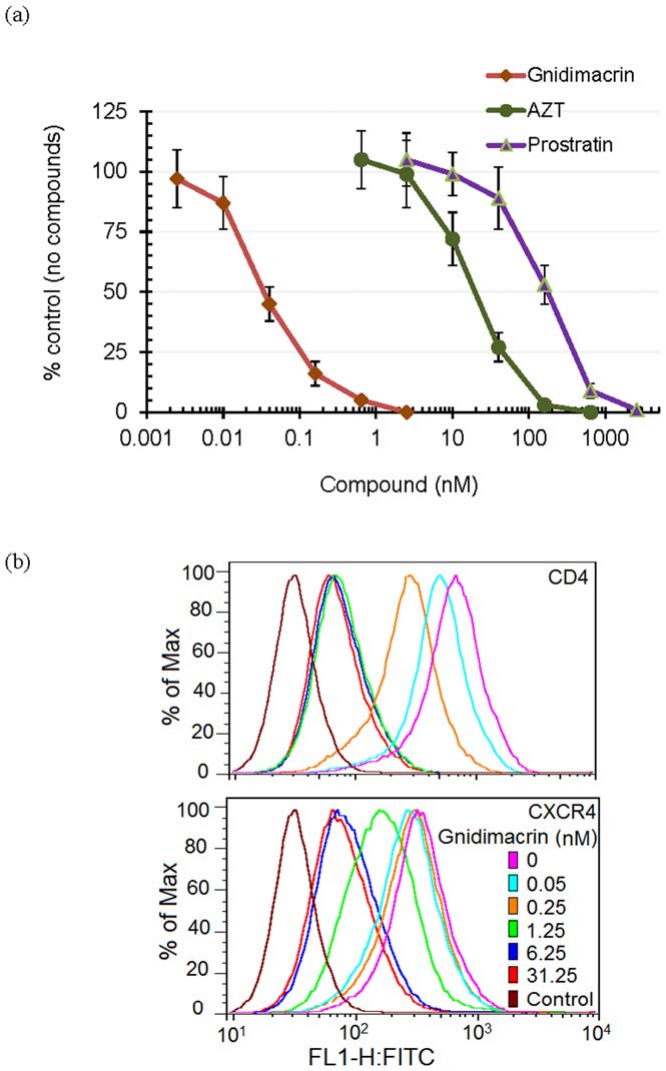
Gnidimacrin inhibited HIV-1 NL4-3 infection at picomolar concentrations. (a) MT4 cells were infected with NL4-3 in the presence of gnidimacrin, AZT, or prostratin for 4 days. The virus replication in the absence of antivirals is defined as 100% (control) virus production. Each data point in the figure represents the mean +/− standard deviation of three independent experiments. (b) Down regulation of CD4 and CXCR4 by gnidimacrin. MT4 cells were treated with gnidimacrin at various concentrations for one day before FACS analysis. The anti-CD4 monoclonal antibody OKT4 and the anti-CXCR4 monoclonal antibody 12G5 were used for the FACS analysis. The color assignment to each assay condition is the same for both FACS panels. A histogram of the FACS data on % receptor down regulation expressed as ratio of mean fluorescence intensity (MFI) is available as additional file 1 (Figure S1).

Gnidimacrin was tested against the ×4 virus NL4-3 infection of MT4 cells at various concentrations. The HIV-1 RT inhibitor AZT, one of the most common antiretroviral drugs used in clinic, and prostratin, the most well studied non-tumour promoting phorborloid for targeting HIV-1 reservoir, were used as controls in the same assays. Gnidimacrin inhibited NL4-3 replication by 50% (EC_50_) at an extremely low concentration of 31 pM (Figure 2a). In comparison, the EC_50_s for AZT and prostratin were 20 nM and 175 nM, respectively. Thus, gnidimacrin was greater than 5,000-fold more potent when compared to prostratin. Gnidimacrin was also greater than 500-fold more potent than AZT in the antiviral assays (Figure 2a).

### Gnidimacrin down regulated CD4 and CXCR4 on MT4 cells

Gnidimacrin was previously reported to be a protein kinase C agonist (13). It has been shown that protein kinase C agonists, such as prostratin, can down regulate CD4 and CXCR4 [20]. To determine whether down regulation of CD4 and CXCR4 is responsible for the anti-HIV-1 activity, MT4 cells were treated with various concentrations of gnidimacrin for one day. The surface expressions of CD4 and CXCR4 on MT4 cells were subsequently analysed with a fluorescence activated cell sorter (FACS). The results indicated that gnidimacrin down regulated both CD4 and CXCR4 expression on MT4 cells in a dose dependent manner (Figure 2b). CD4 is relatively sensitive to gnidimacrin when compared to CXCR4. Gnidimacrin down regulated CD4 by approximately 90% at 1 nM. Higher concentrations of the compound did not further increase CD4 down regulation (Figure 2b; additional file 1: Figure S1). On the other hand, gnidimacrin down regulated CXCR4 by approximately 80% at 5 nM and higher concentrations of the compound did not further increase CXCR4 down regulation.

These results are consistent with the notion that down regulation of the viral receptors could be responsible for the anti-viral activity of gnidimacrin. However, it should be noted that the EC_50_ for gnidimacrin against NL4-3 infection is 31 pM; gnidimacrin at 50 pM only down regulated CD4 and CXCR4 by approximately 25%. It is possible that partial down regulations of both CD4 and CXCR4 result in a synergistic effect to achieve the potent inhibition of NL4-3 infection of MT4 cells. It is also possible that other unknown mechanisms could play a role in the inhibitory activity of gnidimacrin.

### Gnidimacrin exhibited highly selective cytotoxicity against HIV-1 chronically infected cells

**Figure 3:**
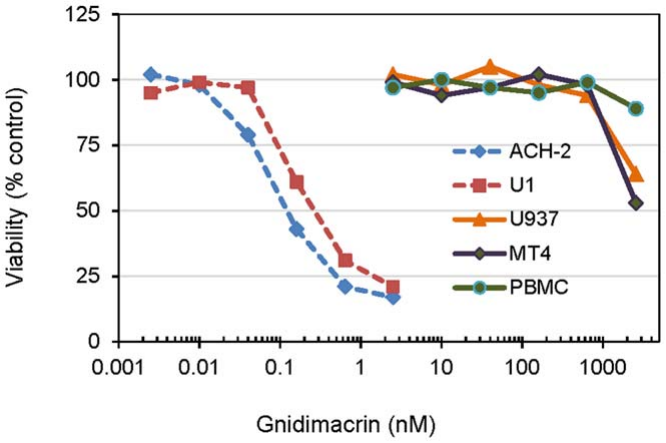
Gnidimacrin exhibited highly selective cytotoxicity against HIV-1 chronically infected cells. ACH-2, U1, and U937 cells were treated with the compounds for three days before the cytotoxicity was determined by using a Promega cell viability assay kit. MT4 and PBMCs were treated with the compounds parallel to the antiviral assays for 4 days and 7 days, respectively. The viability of the cells cultured in the absence of the tested compounds is defined as 100% control. Each data point in the figure represents the average of two independent experiments.

Gnidimacrin is known to have anti-cancer cell activity. Gnidimacrin was shown to selectively inhibit the growth of some lung cancer and leukemic cell lines at sub-nanomolar concentrations [11]. Therefore, the effects of gnidimacrin on the growth of various cell types used in this study including peripheral mononuclear cells (PBMCs), the leukaemia cell line MT4, and the promonocytic cell line U937, were evaluated. The concentration of gnidimacrin that inhibited cell growth by 50% (IC_50_) was greater than 2.5 uM for PBMCs, MT4, and U937 cells (Figure 3). Thus, inhibition of HIV-1 replication by gnidimacrin in MT4 cells was highly selective with a selectivity index (IC_50_/EC_50_) over 8×10^4^. In contrast, the HIV-1 chronically infected cells, U1 and ACH-2, were very sensitive to the cytotoxic effect of gnidimacrin. The IC_50_s of gnidimacrin against ACH-2 and U1 cells are 0.12 nM and 0.25 nM, respectively. Since U1 cells were derived from the monocytic U937 cells, the markedly increased sensitivity of U1 cells to gnidimacrin suggesting that activation of HIV-1 replication by the compound was likely responsible for the cytotoxicity.

### Gnidimacrin inhibited HIV-1 BaL infection of PBMCs at low picomolar concentrations

**Figure 4:**
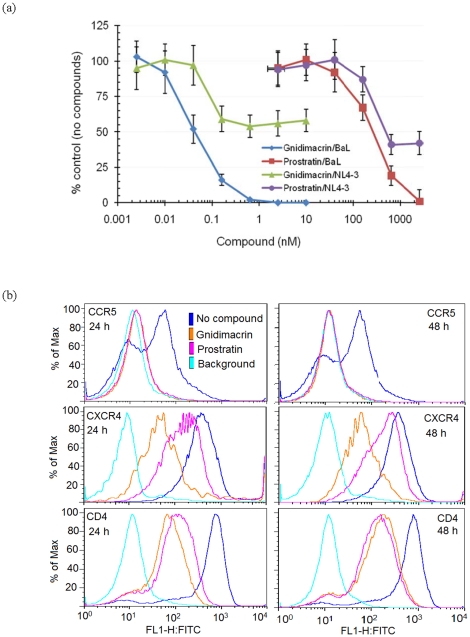
Gnidimacrin inhibited R5 virus infection at picomolar concentrations. (a) HIV-1 BaL and NL4-3 were used to infect CD8-depleted PHA activated PBMCs. The virus replication in the absence of antivirals is defined as 100% control virus production. Each data point in the figure represents the mean +/− standard deviation of three independent experiments. (b) Down regulation of HIV-1 receptors on PBMCs. CD8-depleted PHA activated PBMCs were treated with 1 nM of gnidimacrin or 1 uM of prostratin for 24 hr or 48 hr. Background binding was performed with the same protocol as other conditions, except that primary antibodies were not included in the assay. The color assignment to each assay condition is the same for all six FACS panels. A histogram of the FACS data on % receptor down regulation expressed as ratio of mean fluorescence intensity (MFI) is available as additional file 2 (Figure S2).

HIV-1 uses CD4 and a co-receptor, CCR5 or CXCR4, for entry into susceptible cells. It is well documented that the predominant HIV-1 strains (R5 viruses) isolated from acutely infected individuals use CCR5 for entry [25]. As shown in Figure 2, gnidimacrin exhibited potent inhibitory activity against HIV-1 NL4-3 infection of MT4 cells. NL4-3 is an ×4 virus and MT4 is a leukemic cell line that expresses CXCR4. To determine whether gnidimacrin can inhibit both ×4 and R5 viruses infection of PBMCs, the ×4 virus NL4-3 and the R5 virus HIV-1 BaL were used to infect CD8-depleted PHA activated PBMCs. The anti-HIV-1 protein kinase C agonist prostratin was used as a positive control for the anti-viral activity of gnidimacrin. Unexpectedly, gnidimacrin and prostratin only partially inhibited NL4-3 infection of the CD8-depleted PBMCs. Under the experimental conditions, gnidimacrin was not able to inhibit NL4-3 replication in PBMCs by more than 50% (Figure 4a). In contrast, gnidimacrin potently inhibited HIV-1 BaL infection of the PBMCs by 50% at 42 pM. A similar pattern of inhibition of HIV-1 BaL was observed when prostratin was used in the assay, except that prostratin was approximately 5,000-fold less potent than gnidimacrin (Figure 4a).

### Gnidimacrin down regulated CD4, CCR5, and CXCR4 on PBMCs

To determine whether the anti-HIV-1 activity is correlated with receptor down regulation on PBMCs, the HIV-1 receptors on CD8-depleted PHA activated PBMCs were analysed with a fluorescence activated cell sorter (FACS). All three HIV-1 receptors, CD4, CXCR4, and CCR5 were down regulated in the presence of 1 nM gnidimacrin or 1 uM of prostratin (Figure 4b). Gnidimacrin and prostratin exhibited a similar pattern of HIV-1 receptor down regulation, except that gnidimacrin was at least a thousand fold more potent than prostratin. CCR5 was especially sensitive to gnidimacrin in that down regulation of CCR5 approached 100% one day after gnidimacrin treatment (Figure 4b; additional file 2: Figure S2). CCR5 remained at background level 48 hr after gnidimacrin treatment. The strong down regulation of CCR5 could explain why the R5 virus HIV-1 BaL was extremely sensitive to gnidimacrin.

Similar to that observed on MT4 cells, 1 nM of gnidimacrin down regulated CD4 on the PBMCs by approximately 90%. On the other hand, CXCR4 on PBMCs was more sensitive to gnidimacrin down regulation than that on MT4 cells (Figure 4b; additional file 2: Figure S2). Yet, the ×4 virus NL4-3 infection of MT4 was very sensitive to gnidimacrin with an EC_50_ at 31 pM, whereas NL4-3 infection of PBMCs was only partially inhibited by gnidimacrin. The mechanism responsible for this discrepancy is not clear. Nevertheless, HIV-1 infection of PBMCs reflects in *vivo* HIV-1 infection more closely than the MT4 assay. Therefore, the PBMC assay was used to further evaluate the effect of gnidimacrin on HIV-1 primary isolates.

### Gnidimacrin inhibited R5 HIV-1 primary isolates at low picomolar concentrations

HIV-1 is a highly mutable virus with a highly diverse genomic sequence. Therefore, it is critical for an anti-viral agent to have a broad inhibitory activity against various HIV-1 primary isolates. A panel of R5 and ×4 HIV-1 strains from clade A to D was tested for their sensitivity to gnidimacrin in a PBMC assay. The majority of these HIV-1 strains were from an international panel of HIV-1 isolates representing major globally prevalent strains of genetically and biologically characterized HIV-1 isolates [26]. Regardless of their genetic subtypes, all of the twelve tested R5 HIV-1 strains were very sensitive to gnidimacrin with EC_50_ ranging from 1 pM to 50 pM (Table 1). The average EC_50_ of gnidimacrin is approximately 9 pM against 12 R5 viruses. In contrast, gnidimacrin did not effectively inhibit the four ×4 viruses and the dual tropic virus DH012. Similar to the inefficient inhibition of NL4-3 shown in Figure 4, gnidimacrin was not effective against the tested ×4 primary isolates. None of the four tested ×4 and the dual tropic viruses was inhibited by more than 50% at a gnidimacrin concentration as high as 1 uM under the experimental conditions.

**Table 1:** Gnidimacrin inhibited R5 viruses at low picomolar concentrations.

HIV-1 strains	Clade	Gnidimacrin EC_50_ (pM)	Co-receptor
KNH1088	A	3.1	CCR5
CM240/GS 022	CRF01_AE	3.5	CCR5
CAM0002BBY	CRF02_AG	0.71	CCR5
Ba-L (85US_Ba-L)	B	42.5	CCR5
33931N	B	5.3	CCR5
BX08	B	3.7	CCR5
YU-2	B	4.5	CCR5
HIV-1 JRCSF	B	20.5	CCR5
873 (90US_873)	B	15.5	CCR5
SE364/GS 015	C	3.2	CCR5
20635-4	C	1.3	CCR5
A07412M1	D	12.8	CCR5
NI1052M1	CRF01_AE	P*	CXCR4
BK132/GS 009	B	P*	CXCR4
BZ 167 (GS 010)	B	P*	CXCR4
NL4-3	B	P*	CXCR4
DH012	B	P*	CCR5/CXCR4

CD8 depleted PHA activated PBMCs were infected with HIV-1 for 7 days. The EC_50_s in the table represent the average of two independent experiments. P* denotes partial inhibition of the virus by gnidimacrin.

### Protein kinase C beta plays a key role in the anti-HIV-1 activity of gnidimacrin

**Figure 5:**
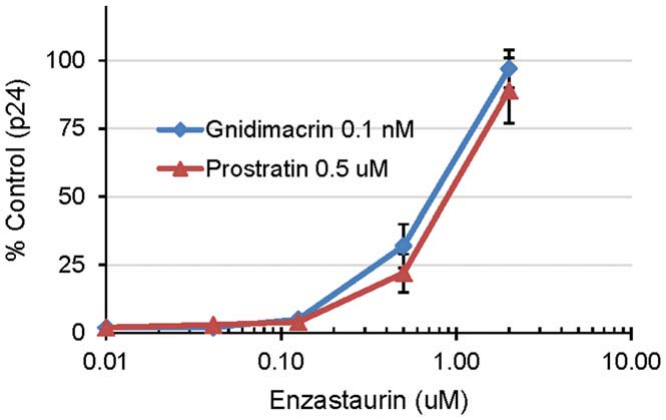
Enzastaurin abrogated the anti-HIV-1 activity of gnidimacrin and prostratin. CD8-depleted PHA activated PBMCs were treated with enzastaurin at indicated concentrations for 2 hours. The enzastaurin treated PBMCs were then infected with HIV-1 BaL in the presence of various concentrations of enzastaurin and 0.1 nM of gnidimacrin or 0.5 uM of prostratin. The culture supernatants were assayed for p24 on day 7 post infection. The virus replication (p24) in the presence of enzastaurin without gnidimacrin or prostratin is defined as 100% control. Each data point in the figure represents the mean +/− standard deviation of three independent experiments.

The protein kinase C beta II was reported to be responsible for the anti-cancer cell activity of gnidimacrin [13]. To test whether it plays a role in the anti-HIV-1 activity of gnidimacrin, a protein kinase C beta selective inhibitor enzastaurin [27] was used to determine whether gnidimacrin targets the enzyme for the potent anti-HIV-1 activity. Enzastaurin at various concentrations was used to antagonize the antiviral activity of gnidimacrin against HIV-1 BaL infection of PBMCs. The anti-HIV-1 activity of 0.1 nM gnidimacrin or 0.5 uM of prostratin was determined in the presence of increasing concentrations of enzastaurin. HIV-1 replication was strongly inhibited by gnidimacrin or prostratin in the absence of enzastaurin (Figure 5). Enzastaurin alone did not affect HIV-1 replication or cell viability under the experimental conditions. Enzastaurin at 2 uM was able to fully abrogate the anti-HIV-1 activity of gnidimacrin or prostratin (Figure 5). This result suggests that protein kinase C beta is the main protein kinase C isozyme responsible for the anti-HIV-1 activity of gnidimacrin in PBMCs.

## Discussion

The results of this study demonstrate that the daphnane diterpene gnidimacrin activated HIV-1 production from chronically infected cells, inhibited HIV-1 R5 virus infection of PBMCs, and killed HIV-1 chronically infected cells at picomolar concentrations. Several other protein kinase C agonists were shown to activate HIV-1 replication and down regulate HIV-1 receptors at nanomolar concentrations. These protein kinase C agonists include prostratin, P-13S, DPP, I3A, SJ23B, and bryostatin [15]–[20], [28]–[30]. Most of these compounds are natural products isolated from plants. Among these compounds, prostratin was the best studied for its potential as an adjuvant therapeutic with HAART to eradicate HIV-1 from its latent reservoirs. Successful semi-synthesis of prostratin has further raised the interest of developing this compound for potential HIV-1 eradication [31]. Thus, prostratin was chosen as a control in this study for its regulatory effect on HIV-1 replication.

The results of this study clearly indicate that gnidimacrin is at least 1,000 fold more effective than prostratin in activation of HIV-1 replication in the latently infected ACH-2 and U1 cells. The effective doses for HIV-1 activation were equivalent to the cytotoxic doses of both compounds. In contrast, U937, the parental cell line of U1, was at least 1,000 fold less sensitive to the cytotoxic effect of gnidimacrin. The differential sensitivity between U1 and U937 to gnidimacrin suggests that activation of HIV-1 replication could potentially eliminate latently infected cells. Gnidimacrin was also at least 1,000 fold more potent than prostratin in inhibiting HIV-1 infection. Gnidimacrin was particularly effective against R5 virus infection of PBMCs. The average EC_50_ for gnidimacrin against the 12 tested R5 viruses was approximately 9 pM. Such a potent inhibitory activity is likely sufficient to inhibit the R5 viruses purged from latent viral reservoirs.

Inhibition of R5 HIV-1 strains by gnidimacrin correlated with down regulation of CCR5 on PBMCs (Figure 4b). CCR5 was down regulated to background level on PBMCs, whereas CD4 and CXCR4 were only partially down regulated by gnidimacrin. Although the ×4 virus NL4-3 replication in MT4 cells is very sensitive to gnidimacrin, ×4 virus replication in PBMCs was only partially inhibited by gnidimacrin and prostratin. Down regulation of HIV-1 receptors does not explain this discrepancy because similar partial down regulation of CD4 and CXCR4 was observed on both PBMCs and MT4 cells (Figure 2b, Figure 4b). One possible explanation is that a small fraction of CD4 and CXCR4 on PBMCs, but not on MT4 cells, are sufficient for HIV-1 infection; as a result, partial down regulation of CD4 and CXCR4 potently inhibits HIV-1 infection of MT4 cells but is not effective against ×4 virus infection of PBMCs.

Like gnidimacrin, prostratin was able to completely inhibit R5 virus, but only partially inhibited ×4 virus infection of PBMCs in this study (Figure 4). The fact that gnidimacrin was ineffective against all tested five viruses that use CXCR4 for entering PBMCs further support the notion that CCR5 down regulation is a key mechanism responsible for the potent anti-R5 virus activity. Although the anti-HIV-1 activity of prostratin in cell lines is well documented, the effect of prostratin on HIV-1 infection of PBMCs is not conclusive. Prostratin was shown to inhibit both R5 and ×4 virus infection of activated PBMCs [32]. In contrast, it has also been shown that prostratin and the ingenol I3A were inactive against NL4-3 (an ×4 virus) infection of activated PBMCs [20]. It is possible that inhibition of ×4 virus infection by gnidimacrin is dependent on cell types and the activation states of the cells.

Protein kinase C is a large family with four conventional isozymes PKCα, PKCβI, PKCβII and PKCγ; four novel isozymes, PKCδ, -ε, -η, and -θ; the atypical isozymes PKCζ and PKCι [33]. Enzastaurin is a selective inhibitor for PKCβI and PKCβII [27]. The strong antagonistic effect of enzastaurin on the anti-HIV-1 activity of gnidimacrin suggests that protein kinase C beta is the major protein kinase C isozyme responsible for the potent anti-HIV-1 activity. We propose that gnidimacrin inhibits HIV-1 by activating protein kinase C beta, which in turn strongly down regulates CCR5 and partially internalizes CD4 and CXCR4.

In conclusion, the results of this study suggest that protein kinase C beta is the target of gnidimacrin. Activation of protein kinase C beta by gnidimacrin causes internalization of HIV-1 receptors CD4, CXCR4, and CCR5, which prevents HIV-1 from entering the cells. Gnidimacrin is an extremely potent HIV-1 regulator that activates HIV-1 replication in chronically infected cells and inhibits R5 HIV-1 strains at low picomolar concentrations. The potent dichotomous activities make gnidimacrin an attractive adjuvant therapeutic candidate for the possibility of HIV-1 eradication. Future challenges in studying this potent anti-HIV-1 agent include identification of the specific cellular pathways that impart this extremely potent dichotomous activity of gnidimacrin.

## Supporting Information

Figure S1 **Dose-dependent down regulation of CD4 and CXCR4 on MT4 cells by gnidimacrin.** MT4 cells were treated with gnidimacrin at various concentrations for one day before FACS analysis. The anti-CD4 monoclonal antibody OKT4 and the anti-CXCR4 monoclonal antibody 12G5 were used for the FACS analysis. The level of each receptor on the cell surface was expressed as % control which is defined as 100× (MFI_c_-MFI_b_/MFI_0_-MFI_b_), where MFI_0_ is the mean fluorescence intensity in the absence of a compound, MFI_c_ is the mean fluorescence intensity in the presence of the test compounds, and MFI_b_ is the background mean fluorescence intensity when the primary antibodies were not used in the assays.
(TIF)

Figure S2 **Down regulation of CCR5, CXCR4, and CD4 on PBMCs by gnidimacrin.** CD8-depleted PHA activated PBMCs were treated with 1 nM of gnidimacrin or 1 uM of prostratin for 24 hr or 48 hr. The X-axial labels, such as CCR5-gnidimacrin, denote the relative level of a receptor in the presence of gnidimacrin or prostratin.
(TIF)
