# Supplementary figures and images for: Picomolar Dichotomous Activity of Gnidimacrin Against HIV-1
Source: PLoS One. 2011 Oct 24;6(10):e26677. doi: 10.1371/journal.pone.0026677 (PMC3200356; doi:10.1371/journal.pone.0026677)

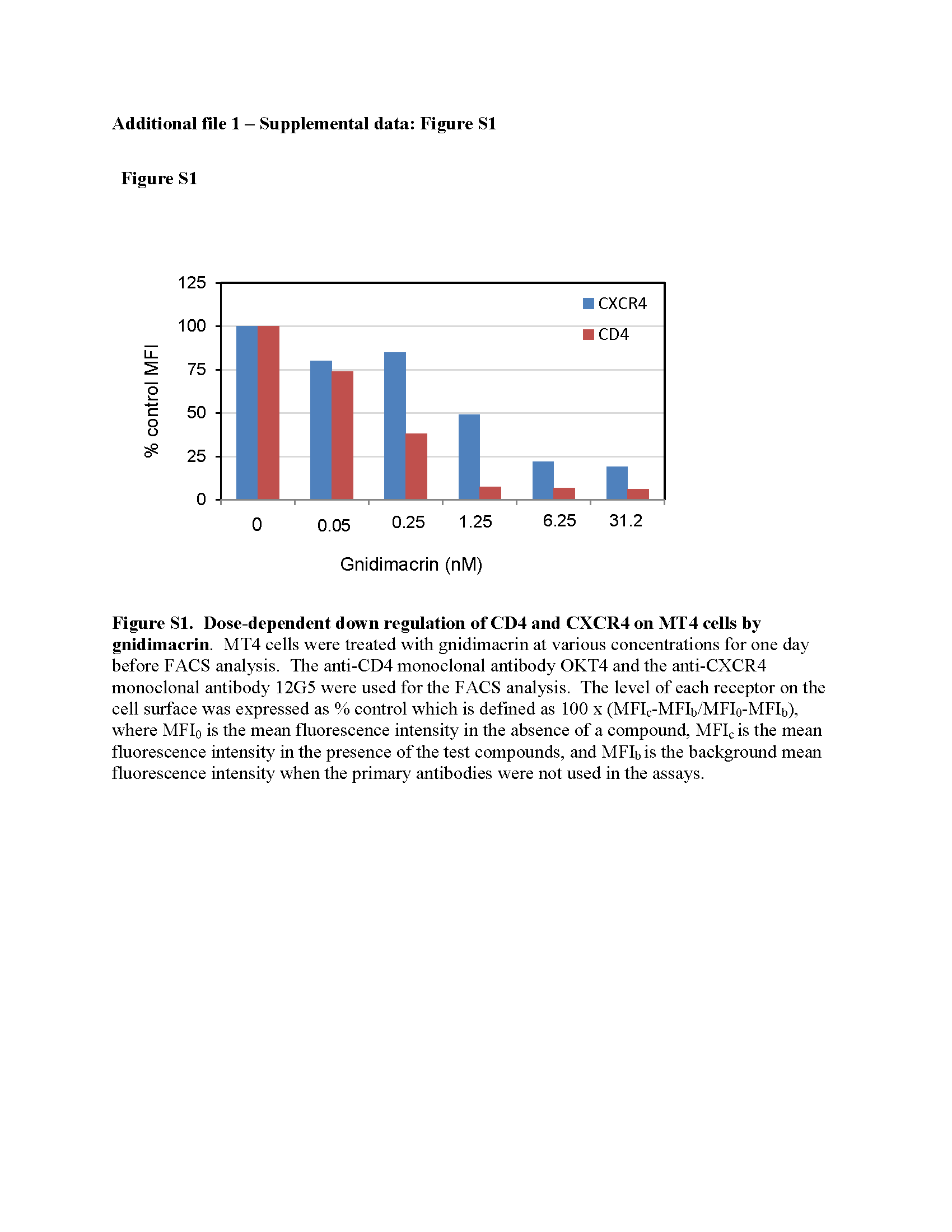

Supplement: Figure S1 — Dose-dependent down regulation of CD4 and CXCR4 on MT4 cells by gnidimacrin. MT4 cells were treated with gnidimacrin at various concentrations for one day before FACS analysis. The anti-CD4 monoclonal antibody OKT4 and the anti-CXCR4 monoclonal antibody 12G5 were used for the FACS analysis. The level of each receptor on the cell surface was expressed as % control which is defined as 100× (MFIc-MFIb/MFI0-MFIb), where MFI0 is the mean fluorescence intensity in the absence of a compound, MFIc is the mean fluorescence intensity in the presence of the test compounds, and MFIb is the background mean fluorescence intensity when the primary antibodies were not used in the assays. (TIF) [file pone.0026677.s001.tif]

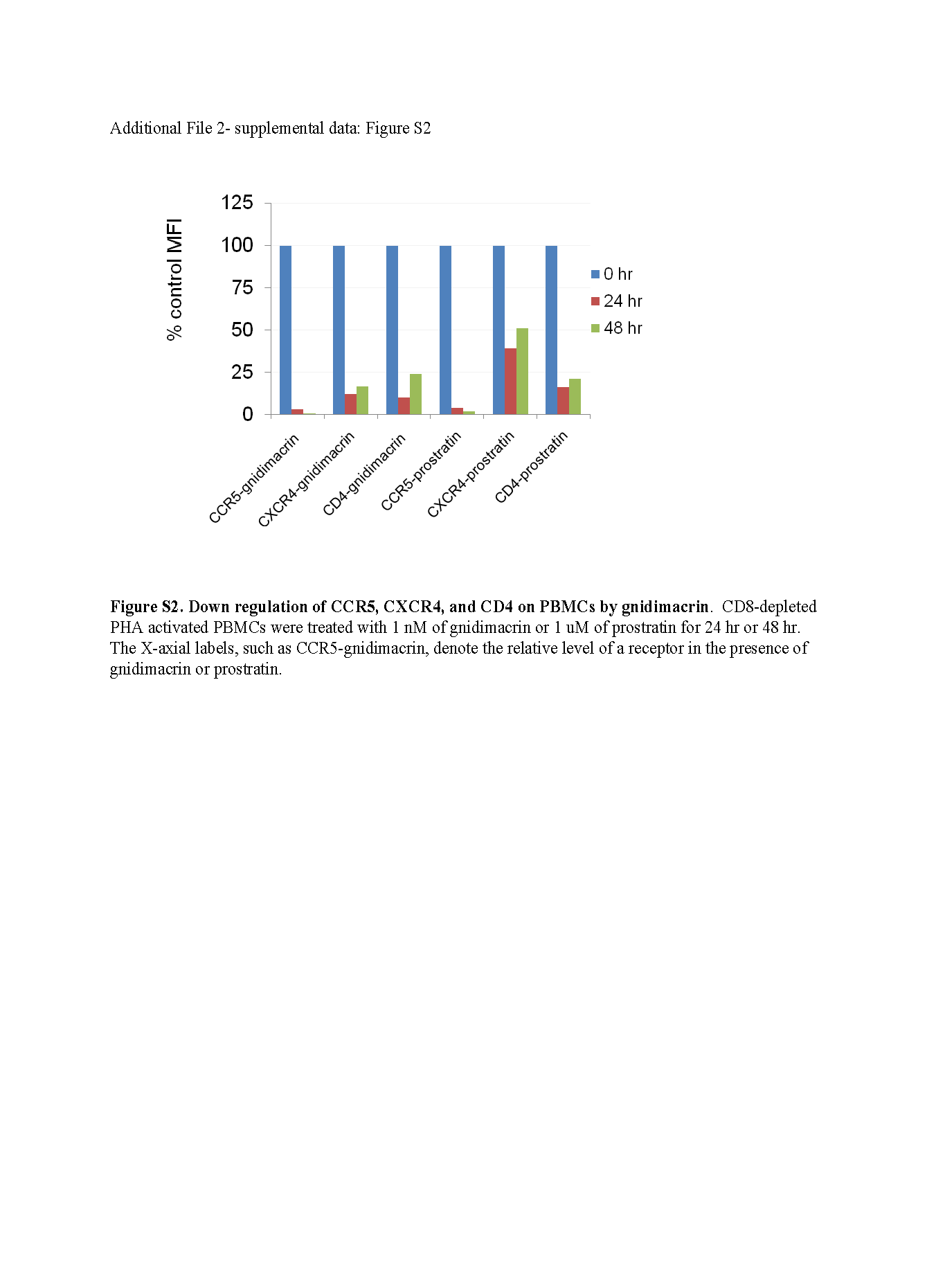

Supplement: Figure S2 — Down regulation of CCR5, CXCR4, and CD4 on PBMCs by gnidimacrin. CD8-depleted PHA activated PBMCs were treated with 1 nM of gnidimacrin or 1 uM of prostratin for 24 hr or 48 hr. The X-axial labels, such as CCR5-gnidimacrin, denote the relative level of a receptor in the presence of gnidimacrin or prostratin. (TIF) [file pone.0026677.s002.tif]
